# Supplementary material for: OpenMM 7: Rapid development of high performance algorithms for molecular dynamics
Source: PLoS Comput Biol. 2017 Jul 26;13(7):e1005659. doi: 10.1371/journal.pcbi.1005659 (PMC5549999; doi:10.1371/journal.pcbi.1005659)
Supplement: S1 Source Code — (ZIP) [file pcbi.1005659.s002.zip › OpenMM-7.0.1-Source/docs/api-c++/generated/OpenMM.Context.html]

Context — OpenMM @OPENMM\_MAJOR\_VERSION@.@OPENMM\_MINOR\_VERSION@ documentation


# Context¶

*class* `OpenMM::``Context`¶
:   A `Context` stores the complete state of a simulation. More specifically, it includes:

    > - The current time
    > - The position of each particle
    > - The velocity of each particle
    > - The values of configurable parameters defined by `Force` objects in the `System`

    You can retrieve a snapshot of the current state at any time by calling `getState()`. This allows you to record the state of the simulation at various points, either for analysis or for checkpointing. `getState()` can also be used to retrieve the current forces on each particle and the current energy of the `System`.

    Methods

    |  |  |
    | --- | --- |
    | `Context` | Construct a new `Context` in which to run a simulation. |
    | `Context` | Construct a new `Context` in which to run a simulation, explicitly specifying what `Platform` should be used to perform calculations. |
    | `Context` | Construct a new `Context` in which to run a simulation, explicitly specifying what `Platform` should be used to perform calculations and the values of platform-specific properties. |
    | `~Context` |  |
    | `getSystem` | Get `System` being simulated in this context. |
    | `getIntegrator` | Get `Integrator` being used to by this context. |
    | `getIntegrator` | Get `Integrator` being used to by this context. |
    | `getPlatform` | Get the `Platform` being used for calculations. |
    | `getPlatform` | Get the `Platform` being used for calculations. |
    | `getState` | Get a `State` object recording the current state information stored in this context. |
    | `setState` | Copy information from a `State` object into this `Context`. |
    | `setTime` | Set the current time of the simulation (in picoseconds). |
    | `setPositions` | Set the positions of all particles in the `System` (measured in nm). |
    | `setVelocities` | Set the velocities of all particles in the `System` (measured in nm/picosecond). |
    | `setVelocitiesToTemperature` | Set the velocities of all particles in the `System` to random values chosen from a Boltzmann distribution at a given temperature. |
    | `getParameters` | Get all adjustable parameters that have been defined by `Force` objects in the `System`, along with their current values. |
    | `getParameter` | Get the value of an adjustable parameter defined by a `Force` object in the `System`. |
    | `setParameter` | Set the value of an adjustable parameter defined by a `Force` object in the `System`. |
    | `setPeriodicBoxVectors` | Set the vectors defining the axes of the periodic box (measured in nm). |
    | `applyConstraints` | Update the positions of particles so that all distance constraints are satisfied. |
    | `applyVelocityConstraints` | Update the velocities of particles so the net velocity of each constrained distance is zero. |
    | `computeVirtualSites` | Recompute the locations of all virtual sites. |
    | `reinitialize` | When a `Context` is created, it may cache information about the `System` being simulated and the `Force` objects contained in it. |
    | `createCheckpoint` | Create a checkpoint recording the current state of the `Context`. |
    | `loadCheckpoint` | Load a checkpoint that was written by `createCheckpoint()`. |
    | `getMolecules` | Get a description of how the particles in the system are grouped into molecules. |

    `Context`(*const* System &*system*, Integrator &*integrator*)¶
    :   Construct a new `Context` in which to run a simulation.

        |  |  |
        | --- | --- |
        | Parameters: |  |

        - **system** – the `System` which will be simulated
        - **integrator** – the `Integrator` which will be used to simulate the `System`

    `Context`(*const* System &*system*, Integrator &*integrator*, Platform &*platform*)¶
    :   Construct a new `Context` in which to run a simulation, explicitly specifying what `Platform` should be used to perform calculations.

        |  |  |
        | --- | --- |
        | Parameters: |  |

        - **system** – the `System` which will be simulated
        - **integrator** – the `Integrator` which will be used to simulate the `System`
        - **platform** – the `Platform` to use for calculations

    `Context`(*const* System &*system*, Integrator &*integrator*, Platform &*platform*, *const* std::map<std::string, std::string> &*properties*)¶
    :   Construct a new `Context` in which to run a simulation, explicitly specifying what `Platform` should be used to perform calculations and the values of platform-specific properties.

        |  |  |
        | --- | --- |
        | Parameters: |  |

        - **system** – the `System` which will be simulated
        - **integrator** – the `Integrator` which will be used to simulate the `System`
        - **platform** – the `Platform` to use for calculations
        - **properties** – a set of values for platform-specific properties. Keys are the property names.

    `~Context`()¶

    *const* System &`getSystem`() *const*¶
    :   Get `System` being simulated in this context.

    *const* Integrator &`getIntegrator`() *const*¶
    :   Get `Integrator` being used to by this context.

    Integrator &`getIntegrator`()¶
    :   Get `Integrator` being used to by this context.

    *const* Platform &`getPlatform`() *const*¶
    :   Get the `Platform` being used for calculations.

    Platform &`getPlatform`()¶
    :   Get the `Platform` being used for calculations.

    State `getState`(int *types*, bool *enforcePeriodicBox* = false, int *groups* = 0xFFFFFFFF) *const*¶
    :   Get a `State` object recording the current state information stored in this context.

        |  |  |
        | --- | --- |
        | Parameters: |  |

        - **types** – the set of data types which should be stored in the `State` object. This should be a union of DataType values, e.g. (State::Positions | State::Velocities).
        - **enforcePeriodicBox** – if false, the position of each particle will be whatever position is stored in the `Context`, regardless of periodic boundary conditions. If true, particle positions will be translated so the center of every molecule lies in the same periodic box.
        - **groups** – a set of bit flags for which force groups to include when computing forces and energies. Group i will be included if (groups&(1<<i)) != 0. The default value includes all groups.

    void `setState`(*const* State &*state*)¶
    :   Copy information from a `State` object into this `Context`. This restores the `Context` to approximately the same state it was in when the `State` was created. If the `State` does not include a piece of information (e.g. positions or velocities), that aspect of the `Context` is left unchanged.

        Even when all possible information is included in the `State`, the effect of calling this method is still less complete than `loadCheckpoint()`. For example, it does not restore the internal states of random number generators. On the other hand, it has the advantage of not being hardware specific.

    void `setTime`(double *time*)¶
    :   Set the current time of the simulation (in picoseconds).

    void `setPositions`(*const* std::vector<Vec3> &*positions*)¶
    :   Set the positions of all particles in the `System` (measured in nm). This method simply sets the positions without checking to see whether they satisfy distance constraints. If you want constraints to be enforced, call `applyConstraints()` after setting the positions.

        |  |  |
        | --- | --- |
        | Parameters: |  |

        - **positions** – a vector whose length equals the number of particles in the `System`. The i’th element contains the position of the i’th particle.

    void `setVelocities`(*const* std::vector<Vec3> &*velocities*)¶
    :   Set the velocities of all particles in the `System` (measured in nm/picosecond).

        |  |  |
        | --- | --- |
        | Parameters: |  |

        - **velocities** – a vector whose length equals the number of particles in the `System`. The i’th element contains the velocity of the i’th particle.

    void `setVelocitiesToTemperature`(double *temperature*, int *randomSeed* = osrngseed())¶
    :   Set the velocities of all particles in the `System` to random values chosen from a Boltzmann distribution at a given temperature.

        |  |  |
        | --- | --- |
        | Parameters: |  |

        - **temperature** – the temperature for which to select the velocities (measured in Kelvin)
        - **randomSeed** – the random number seed to use when selecting velocities

    *const* std::map<std::string, double> &`getParameters`() *const*¶
    :   Get all adjustable parameters that have been defined by `Force` objects in the `System`, along with their current values.

    double `getParameter`(*const* std::string &*name*) *const*¶
    :   Get the value of an adjustable parameter defined by a `Force` object in the `System`.

        |  |  |
        | --- | --- |
        | Parameters: |  |

        - **name** – the name of the parameter to get

    void `setParameter`(*const* std::string &*name*, double *value*)¶
    :   Set the value of an adjustable parameter defined by a `Force` object in the `System`.

        |  |  |
        | --- | --- |
        | Parameters: |  |

        - **name** – the name of the parameter to set
        - **value** – the value of the parameter

    void `setPeriodicBoxVectors`(*const* Vec3 &*a*, *const* Vec3 &*b*, *const* Vec3 &*c*)¶
    :   Set the vectors defining the axes of the periodic box (measured in nm). They will affect any `Force` that uses periodic boundary conditions.

        Triclinic boxes are supported, but the vectors must satisfy certain requirements. In particular, a must point in the x direction, b must point “mostly” in the y direction, and c must point “mostly” in the z direction. See the documentation for details.

        |  |  |
        | --- | --- |
        | Parameters: |  |

        - **a** – the vector defining the first edge of the periodic box
        - **b** – the vector defining the second edge of the periodic box
        - **c** – the vector defining the third edge of the periodic box

    void `applyConstraints`(double *tol*)¶
    :   Update the positions of particles so that all distance constraints are satisfied. This also recomputes the locations of all virtual sites.

        |  |  |
        | --- | --- |
        | Parameters: |  |

        - **tol** – the distance tolerance within which constraints must be satisfied.

    void `applyVelocityConstraints`(double *tol*)¶
    :   Update the velocities of particles so the net velocity of each constrained distance is zero.

        |  |  |
        | --- | --- |
        | Parameters: |  |

        - **tol** – the velocity tolerance within which constraints must be satisfied.

    void `computeVirtualSites`()¶
    :   Recompute the locations of all virtual sites. There is rarely a reason to call this, since virtual sites are also updated by `applyConstraints()`. This is only for the rare situations when you want to enforce virtual sites but

    void `reinitialize`()¶
    :   When a `Context` is created, it may cache information about the `System` being simulated and the `Force` objects contained in it. This means that, if the `System` or Forces are then modified, the `Context` might not see all of the changes. Call `reinitialize()` to force the `Context` to rebuild its internal representation of the `System` and pick up any changes that have been made.

        This is an expensive operation, so you should try to avoid calling it too frequently.

    void `createCheckpoint`(std::ostream &*stream*)¶
    :   Create a checkpoint recording the current state of the `Context`. This should be treated as an opaque block of binary data. See `loadCheckpoint()` for more details.

        |  |  |
        | --- | --- |
        | Parameters: |  |

        - **stream** – an output stream the checkpoint data should be written to

    void `loadCheckpoint`(std::istream &*stream*)¶
    :   Load a checkpoint that was written by `createCheckpoint()`.

        A checkpoint contains not only publicly visible data such as the particle positions and velocities, but also internal data such as the states of random number generators. Ideally, loading a checkpoint should restore the `Context` to an identical state to when it was written, such that continuing the simulation will produce an identical trajectory. This is not strictly guaranteed to be true, however, and should not be relied on. For most purposes, however, the internal state should be close enough to be reasonably considered equivalent.

        A checkpoint contains data that is highly specific to the `Context` from which it was created. It depends on the details of the `System`, the `Platform` being used, and the hardware and software of the computer it was created on. If you try to load it on a computer with different hardware, or for a `System` that is different in any way, loading is likely to fail. Checkpoints created with different versions of OpenMM are also often incompatible. If a checkpoint cannot be loaded, that is signaled by throwing an exception.

        |  |  |
        | --- | --- |
        | Parameters: |  |

        - **stream** – an input stream the checkpoint data should be read from

    *const* std::vector<std::vector<int>> &`getMolecules`() *const*¶
    :   Get a description of how the particles in the system are grouped into molecules. Two particles are in the same molecule if they are connected by constraints or bonds, where every `Force` object can define bonds in whatever way are appropriate to that force.

        Each element lists the indices of all particles in a single molecule. Every particle is guaranteed to belong to exactly one molecule.

High performance molecular simulation on GPUs


### Navigation

- Core Objects
- Forces
- Integrators
- Extras

©2015, Stanford University and the Authors.
|
Powered by Sphinx 1.3.1
& Alabaster 0.7.7
|
Page source
